# Progression of lipase activity and pancreatic lipase immunoreactivity in clinically healthy cats and cats with diet-responsive enteropathy

**DOI:** 10.1177/1098612X251367621

**Published:** 2025-09-28

**Authors:** Freya Moscoso Uribe, Barbara Riond, Francesca Del Chicca, Maja Ruetten, Felix Grimm, Annette Liesegang, Peter H Kook

**Affiliations:** 1 Institute of Animal Nutrition and Dietetics, Vetsuisse Faculty, University of Zurich, Zurich, Switzerland; 2 Clinical Laboratory, Vetsuisse Faculty, University of Zurich, Zurich, Switzerland; 3 Clinic of Diagnostic Imaging, Vetsuisse Faculty, University of Zurich, Zurich, Switzerland; 4 Pathovet, Tagelswangen, Switzerland; 5 Institute of Parasitology, Vetsuisse Faculty, University of Zurich, Zurich, Switzerland; 6 Clinic for Small Animal Internal Medicine, Vetsuisse Faculty, University of Zurich, Zurich, Switzerland

**Keywords:** Pancreatitis, lipase, enteropathy, diet, progression

## Abstract

**Objectives:**

The aim of the present study was to describe the course of lipase activity, pancreatic lipase immunoreactivity (PLI) and clinical findings over time in cats.

**Methods:**

Four clinically healthy cats and two diarrhoeic cats from a research colony aged 2–8 years with normal haematology and serum biochemistry results were followed up with lipase measurements over a total of 12 months in this descriptive study. Lipase activity (LIPCRoche; reference interval [RI] 8–26 U/l) was determined at day 0, and lipase activity and concurrent PLI (Spec fPL; RI 0–4.4 µg/l) were determined at days 19, 47, 54, 221 and 369. All cats were examined weekly. The pancreas and gastrointestinal tract of all cats were examined via ultrasonography.

**Results:**

Lipase activity and PLI in four clinically healthy cats was in the range of 10–283 U/l (median 69) and 1.2–86 µg/l (median 13), respectively. Lipase activity and PLI in two cats with enteropathy was in the range of 16–130 U/l (median 42) and 1.9–36 µg/l (median 8.3). The magnitude and nature of change were always the same for both assays. The correlation between assays was very high (*r*_s_ 0.984; *P* <0.0001). The pancreas was normal on ultrasound in both diarrhoeic cats and two healthy cats, whereas a hypoechoic and enlarged pancreas was found in two clinically healthy cats with persistently increased lipase values. All cats had ultrasonographic evidence of enteropathy. No pattern could be recognised in the temporal lipase progression; only one healthy cat with an ultrasonographically abnormal pancreas had continuously increasing values. Both cats with large bowel diarrhoea were diet-responsive.

**Conclusions and relevance:**

Lipase activity and PLI varied from normal to markedly increased in clinically healthy cats and cats with diet-responsive enteropathy and a normal pancreas on ultrasonography. Both lipase assays yielded virtually identical results. No apparent association between lipase results and clinical or ultrasonographic findings was found. The results illustrate the difficulties clinicians face when trying to assess the significance of lipase levels in cats.

## Introduction

Pancreatitis is a frequent histopathological finding in cats, as shown in necropsy studies.^1,2^ Clinical signs are often vague and non-specific, and do not differ between acute and chronic forms.^3^ Because ultrasonographic and laboratory evidence of pancreatitis correlates poorly,^4^ it is difficult for clinicians to diagnose pancreatitis in cats. Lipase measurement is widely used for the clinical diagnosis of pancreatitis in cats, although the exact lipase level at which pancreatitis is truly present remains unknown. Lipase is commonly measured as a concentration (pancreatic lipase immunoreactivity [PLI]) or an activity using the substrate 1,2-*o*-dilauryl-rac-glycero-3-glutaric acid-(6’-methylresorufin)-ester (DGGR).^4–9^ Both assays correlate highly^2,4,7,8^ and perform equally well when compared with a standardised histological examination of the entire pancreas and pancreatic ultrasonographic assessment.^2,4^

Increased lipase activity and PLI concentration have also been reported in cats without a clinical diagnosis of pancreatitis.^5,10^ It was speculated this is either due to a secondary pancreatopathy induced by a primary non-pancreatic disease or due to pre-existing chronic pancreatitis.^10^ No information is available on the progression of lipase activity and PLI over time in clinically healthy adult cats or in cats with non-pancreatic disease. When diarrhoea occurred in two cats in a group of previously healthy cats, and laboratory testing revealed increased lipase activities, we began monitoring the progress of these cats. Our aim was to describe the course of lipase activity and PLI over time in cats without suspected pancreatitis. This felt important as there is uncertainty among veterinarians about the magnitude of lipase increases in cats without suspicion of pancreatitis.

## Materials and methods

### Cats

Six intact domestic shorthair cats, including four female cats (cats 1, 3, 4 and 6) and two male cats (cats 2 and 5), with a median age of 8 years (range 2–8) and a median body weight of 3.86 kg (range 2.72–5.29) (Table 1), from the research colony of the Institute of Animal Nutrition and Dietetics are described. The cats’ median body condition score (BCS) was 5 (range 4–6). Cats were housed in groups, separated by sex, in an indoor-and-outdoor, environmentally enriched facility and supervised daily by veterinarians and technicians. The purpose of the research colony is feeding trials. No cat had been enrolled in experiments over the preceding 24 months. Cats were fed ad libitum. One cat (cat 3 [blue], aged 8 years, weighing 4.12 kg, BCS 6) was fed restrictively (80% of the calculated recommended allowance according to National Research Council guidelines)^11^ to enable approximation to ideal BCS. All cats were regularly vaccinated.^12^

**Table 1:** Weight development (kg) at time points of lipase measurements in four clinically healthy cats (cats 2, 3, 5 and 6) and two initially diarrhoeic cats (cats 1 and 4) that were diagnosed with diet-responsive enteropathy

Cat	t1 (day 0)	t2 (day 19)	t3 (day 47)	t4 (day 54)	t5 (day 102)	t5 (day 221)	t6 (day 369)
1	3.30	3.41	3.42	3.42	3.42	3.40	3.51
2	5.29	5.24	5.19	5.46	5.69	5.20	4.95
3	4.12	3.98	4.06	4.10	4.12	4.20	4.12
4	2.72	2.68	2.99	3.11	3.00	3.10	3.10
5	4.70	4.49	4.68	4.82	5.02	4.90	4.88
6	3.60	3.60	3.65	3.72	4.07	4.40	4.26

## Procedures

In October 2023, blood sampling was performed as part of a checkup for a planned feeding trial. Evaluations were prompted by the onset of large bowel diarrhoea in 2/6 cats (cats 1 [pink] and 4 [green], both aged 8 years; faecal scores 6 and 7, respectively).^13^ A new maintenance dry food (dry food 1, Table 2) had previously been introduced in the cat colony. Faecal scores of the other four cats were consistently 2. Except for the cat fed restrictively (cat 3, blue), all cats had a stable body weight documented for a minimum of 6 months. All cats were observed daily and weighed weekly. The study was approved by the Cantonal Veterinary Office of Zurich and conducted in accordance with guidelines established by the Animal Welfare Act of Switzerland (No ZH162/2022).

**Table 2:** Nutritional composition of different dry foods and macronutrient energetic contribution

	Dry food 1*	Dry food 2^†^	Dry food 3^‡^	Dry food 4^§^
Energy (MJ ME)	1.6	1.6	1.6	1.5
Energy density (MJ/100 g DM)	1.7	1.7	1.7	1.6
Energy (kcal ME)	383	394	385	364
Crude protein (g)	34.0	33.8	25.0	34.0
Protein (% ME)	35.5	35.0	26.0	37.4
Nitrogen-free extract (g)	38.0	34.1	42.0	42.0
Nitrogen-free extract (% DM)	41.3	36.1	44.2	44.7
Nitrogen-free extract (% ME)	39.7	35.3	43.6	46.2
Fat (g)	13.0	17.4	17.0	9.0
Fat (% DM)	14.1	18.5	17.9	9.6
Fat (% ME)	30.5	18.0	39.7	23.7
Fibre (g)	1.6	4.3	3.7	1.8
Fibre (% DM)	1.7	4.5	3.9	1.9
Fibre (% ME)	0.84	1.1	2.03	0.99

Values are absolute amounts (g or kcal) and percentage contributions to metabolisable energy (% ME). The diets varied in their macronutrient distribution, predominantly with differences in nitrogen-free extract and fat content

* CatCat Classic adult

† Hill’s Prescription Diet Gastrointestinal Biome

‡ Royal Canin Anallergenic

§ Vet-Concept Cat Intestinal Low Fat

DM = dry matter; kcal = kilocalories; MJ = megajoules

The study timeline is expressed as time points (t) at blood sampling events, paired with observation days of lipase activity and PLI, as well as dietary management (day 19 = initiation of trial diet).

On day 0, upon first health-check (t1), a complete blood count (CBC; Sysmex XN-1000 including a manual blood smear review) and serum biochemistry profile were performed in all cats. Recorded biochemistry variables (Cobas c501; Roche Diagnostics) consisted of alkaline phosphatase activity, aspartate aminotransferase activity, alanine aminotransferase activity, lipase activity and concentrations of glucose, total protein, albumin, bilirubin, creatinine, urea, triglycerides, cholesterol, calcium, phosphate, sodium, chloride and potassium. Serum amyloid A (SAA) concentrations (Turbidimetric immunoassay MAST Eiken; Eiken)^14^ were also measured. Lipase activity was measured using a DGGR-based assay (LIPC, Roche on Cobas; Roche Diagnostics, reference interval (RI) 8–26 U/l).^6^ PLI concentrations were not initially measured.

## Results

### Laboratory results at t1

CBC and serum biochemistry results were all normal except for the lipase activities. Lipase activity was minimally increased in cat 4 (30 U/l; RI 8–26) and clearly increased in the other five cats, in the range of 82–139 U/l, with a median lipase activity of 85 U/l for all six cats. SAA concentrations were within the RI (0.3–3.3 mg/l) in five cats and increased (41.9 mg/l) in one clinically healthy cat (cat 3, blue).

### Parasitological examination at t1

Faecal samples were pooled by colony group and examined by routine flotation method; they were also tested for *Giardia* species (PCR). All results were negative. Cats were dewormed with Milbemycin oxime and praziquantel preparation (Milpro M; Virbac).

### Diets

A feeding trial was initiated at t2 (day 19) with the introduction of a trial diet (dry food 2), a veterinary therapeutic gastrointestinal diet. Dietary information is provided in Table 2.

### Ultrasonography examination

The pancreas and intestinal tract were examined in a standardised manner by an experienced board-certified radiologist on day 57 (t4) using a GE Logiq E10 machine (GE HealthCare) with different probes (curvilinear array, linear array and hockey stick, depending on region of interest), with frequencies in the range of 5–18 MHz. The pancreas was evaluated for echogenicity, homogeneity and thickness. In addition, small and large intestinal wall thicknesses and presence of layering were assessed. Measurements were performed on the duodenum (caudal to the papilla), jejunum, ileum (the most aboral segment, when possible between folds)^15^ and colon descendens, all imaged parallel to the long axis of the intestinal segment. The presence of mesenteric lymphadenopathy was also recorded. The ultrasonographer was blinded to all clinical and laboratory information.

### Ultrasonographic assessment of the pancreas and gastrointestinal tract

All pancreatic parts (body, right and left limb) from all six cats were examined. The pancreas was ultrasonographically normal in 4/6 cats. The pancreas was hypoechoic and enlarged with a normal peripancreatic mesentery in clinically healthy cats 3 (blue) and 6 (red) (Table 3). Cats 3 and 6 also had increased total wall thicknesses, and cat 3 had thickened lamina muscularis of the small intestines (Table 4).^15,16^ Compared with a cutoff of 2.8 mm,^17^ six cats had at least one abnormally thickened small bowel segment. A thickened lamina muscularis and thickened colonic wall was found in one diarrhoeic cat (cat 4, green). Intestinal and pancreatic ultrasonographic findings were mildest in cat 1, the most severely clinically affected (higher defecation frequency, occasional mucus admixture).

**Table 3:** Ultrasonographic assessment of the pancreas of four clinically healthy cats (cats 2, 3, 5 and 6) and two diarrhoeic cats (cats 1 and 4) at t4 (day 57)

Cat	Pancreatic size	Echogenicity compared with mesentery	Echogenicity of the peripancreatic mesentery	Final assessment
1	Normal	Isoechoic	Normal	Normal pancreas
2	Normal	Isoechoic	Normal	Normal pancreas
3	Enlarged	Hypoechoic	Normal	Low-grade pancreatopathy/pancreatitis
4	Normal	Isoechoic	Normal	Normal pancreas
5	Normal	Hypoechoic	Normal	Normal pancreas
6	Enlarged	Hypoechoic	Normal	Low-grade pancreatopathy/pancreatitis

Parameters evaluated include pancreatic size, echogenicity relative to the surrounding mesentery, echogenicity of the peripancreatic mesentery and final ultrasonographic interpretations

**Table 4:** Ultrasonographic measurements and assessment of the intestinal tract of four clinically healthy cats (cats 2, 3, 5 and 6) and two cats with diet responsive enteropathy (cats 1 and 4) at t4 (day 57)

Cat	Duodenum (mm)	Papilla (mm)	Jejunum (mm)	Ileum aboral (mm)	Colon descendens (mm)	Final assessment
1	2.5	1.7	2.3	3.3	1.2	Normal gastrointestinal tract
2	2.5	3.3	2.4	3.6	1.6	Low-grade diarrhoea/colitis
3	2.3	2.9	3.0	4.0	0.9	Slight thickening of the lamina muscularis (duodenum, jejunum, colon)
4	2.5	2.2	2.9	3.0	1.9	Slight thickening of the lamina muscularis duodenum, jejunum Colitis
5	2.7	2.7	2.4	3.2	0.8	Multifocal mild lymphadenomegaly
6	2.8	4.1	2.8	2.9	1.0	Multifocal mild lymphadenomegaly

### Laboratory results at t2

**Figure 1:**
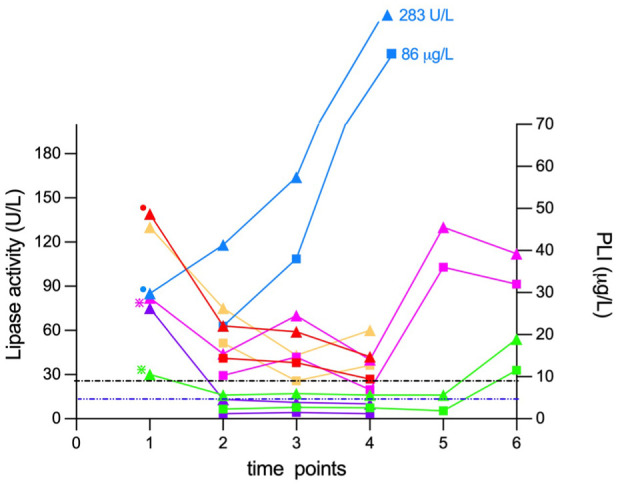
Progression of lipase activity (triangles) and pancreatic lipase immunoreactivity (PLI) concentration (squares) in four clinically healthy cats (yellow, blue, purple and red symbols) and two cats with diet-responsive diarrhoea (pink, green). The dots at t1 illustrate cats with an ultrasonographic diagnosis of pancreatitis; the asterisks illustrate cats with diarrhoea as the only clinical sign. The black dashed line represents the upper reference interval (RI) limit of lipase activity (26 U/l) and the blue dashed line represents the upper RI limit of the PLI test (0–4.4 µg/l)

At 19 days after baseline checkup (t2), the same biochemistry panel was repeated in all cats and a trial diet was initiated. In addition, PLI (Spec fPL, RI 0–4.4 µg/l) was measured (IDEXX Diavet). Progression of lipase activity and PLI concentrations can be seen in Figure 1. Lipase activity had increased in one cat (cat 3, blue) and decreased in the other five cats but remained higher than the RI in three cats. In two cats (cats 4 [green] and 5 [purple]), lipase activity had normalised within the RI.

### Further development and clinical signs

**Figure 2:**
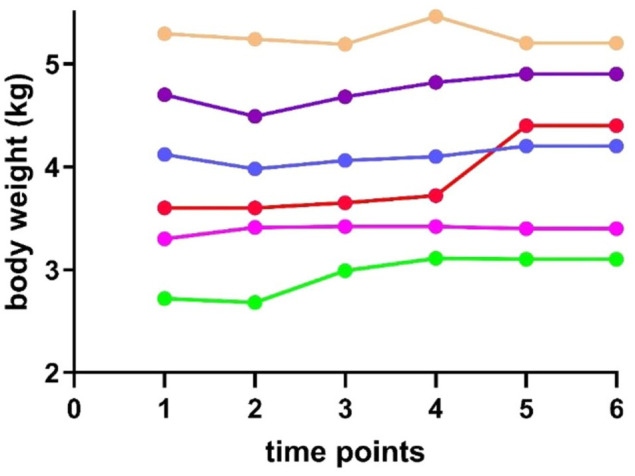
Weight progression of six cats over the study period based on Table 1. Each cat is represented by a distinct colour (pink = cat 1; yellow = cat 2; blue = cat 3; green = cat 4; purple = cat 5; red = cat 6). Measurements were taken at the following time points: t1 (day 0, baseline), t2 (day 19, after initial dietary change), t3 (day 47), t4 (day 54, pre-ultrasound assessment), t5 (day 221 for cat 4, day 102 for cat 1, after dietary intervention) and t6 (day 369, final follow-up). No relevant weight fluctuations were observed at any time point

Four cats (cats 2 [yellow], 3 [blue], 5 [purple] and 6 [red]) remained clinically healthy throughout the study (Table 1, Figure 2). Diarrhoea persisted in cats 1 (pink) and 4 (green) despite the dietary change to a trial diet (dry food 2) (Table 2). Both cats are described in more detail below.

### Further progression of lipase activities and PLI concentrations

On days 47 (t3) and 57 (t4), lipase activity and PLI were rechecked in all cats.

In two cats (cats 4 [green] and 5 purple]), lipase activity remained within the RI. PLI concentrations in these two cats were also within the RI.

Median lipase activity of the remaining four cats, whose values were still increased at t2, was 69 U/l (range 44–118) and their median PLI concentration was 16.2 µg/l (range 10.3–22).

Additional lipase activity and PLI rechecks were available for the two cats with diarrhoea (cat 1 [pink]: days 102 [t5] and 369 [t6]; cat 4 [green]: days 221 [t5] and 369 [t6]).

At t6, both lipase activity and PLI had increased again in both cats (Figure 1). SAA concentrations were within the RI in both cats at t4, t5 and t6.

The magnitude and nature of lipase changes were always the same for both assays (Figure 1). When the results of all 22 paired lipase activities and PLI concentrations were analysed, a very strong significant correlation was found (Spearman’s rho coefficient *r*_s_ = 0.993; *P* <0.0001). Cat 3 (blue) was the cat with the highest lipase activity and PLI values throughout the study period, as well as having ultrasonographic evidence of pancreatitis.

### Further results in two cats with diarrhoea as the only clinical sign

Cats 1 and 4 had recurring large bowel diarrhoea on the trial diet (dry food 2). Defecation frequency was increased (5–6 times daily), small amounts of faeces were passed and tenesmus was occasionally observed. Both cats continued to be lively and alert, with normal responsiveness to stimuli and good appetite. No other clinical signs were noted.

**Figure 3:**
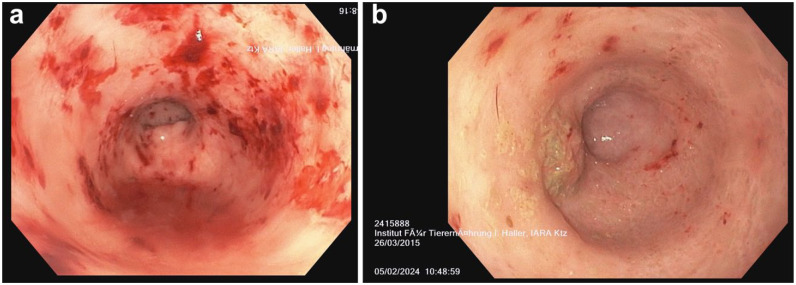
(a) Endoscopic view (Olympus GIF-H185) of the descending colon in cat 1. Multiple colonic erosive and ulcerative lesions with multifocal haemorrhages were observed. The entire colon was affected, with the descending colon appearing more severely affected. (b) Endoscopic view (Olympus GIF-H185) of the ascending colon in cat 1. Multiple erosive lesions are seen, less severe compared with the descending colon

After a PCR test for *Tritrichomonas foetus* (PCR, fresh faecal sample) was negative, upper and lower gastrointestinal endoscopy was performed in cat 1 at t5. Multiple colonic erosive lesions with multifocal haemorrhages were observed. The entire colon was affected, the colon descendens appeared more severely affected (Figure 3). The severity of endoscopic colonic lesions was unexpected. The oesophagus, stomach, duodenum, ileum and distal jejunum were macroscopically normal. Biopsies from all examined locations were examined by a board-certified pathologist.^18^ Mild chronic follicular gastritis, mild chronic lymphoplasmacytic enteritis with mild fibrosis in duodenal and jejunal biopsies, and moderate chronic neutrophilic and ulcerative colitis were found. Colonic Periodic Acid–Schiff and Steiner silver stainings were negative for *Anaerobiospirillum* species.^19^

After endoscopy at t4, cat 1 was treated with budesonide (1 mg q24h for 7 days) and her diet was changed to dry food 3 (Table 2). Normalisation of faecal score (score 2–3) was observed after 14 days. Formed stools (faecal score 3, occasionally score 4) were passed once daily over the following 12 weeks, until the last lipase measurement at t6. No other clinical signs were noted and body weights remained stable (Table 1, Figure 2) until the time of manuscript preparation (day 402).

Cat 4 (green) was adopted by a veterinarian after t4. As diarrhoea persisted (faecal score 5–6), her diet was changed to dry food 4 (Table 2) on day 92, along with probiotic supplementation (SivoMixx Slab51 Drops; Ormendes); however, faecal consistency deteriorated (score 7). Repeated blood work (CBC and biochemistry), faecal screening for enteropathogenic bacteria that included faecal cultures for *Salmonella* species, *Yersinia enterocolitica* and *Campylobacter* species, as well as a general fungal culture and PCR testing for feline coronavirus (Feline Diarrhea Panel; IDEXX), and PCR testing for *T foetus* revealed no abnormalities. A 2-week trial of prednisolone (0.5 mg/kg q24h) resulted in no improvement. On day 112, the diet was changed again to the trial diet (dry food 2); psyllium husks (1.7 g q24h) were introduced, resulting in improved stool consistency (faecal score 4).

## Discussion

We present for the first time the progression of lipase activity and PLI concentration over time in a group of cats without suspicion of pancreatitis – namely, clinically healthy cats and diarrhoeic cats – which were diagnosed with diet-responsive enteropathy. There was no discernible difference between both assays; additional PLI determination provided no added diagnostic value. Our findings contradict the assertation that DGGR-based lipase activity is less specific than PLI concentrations.^20^ Moreover, neither lipase results nor clinical signs correlated with ultrasonographic pancreatic findings. Increased lipase activity and PLI concentration was also observed in diarrhoeic cats with normal pancreatic ultrasound, which had a clinical diagnosis of diet-responsive enteropathy. We believe the results of the present study reflect what we have observed in the feline patients at our hospital, as well as findings recently published in the literature^10^ – namely, various points that we will discuss in the following.

A recent retrospective study reported increased lipase activities in hospitalised cats with a variety of non-pancreatic diseases but without a clinical diagnosis of pancreatitis.^10^ Those results illustrate how difficult it is for clinicians to understand when an increased lipase result is due to an underlying pancreatitis that also causes the presenting clinical signs. Subclinical chronic pancreatopathy can cause hyperlipasaemia in cats, but the presenting clinical signs may originate from an extrapancreatic disease. It is unknown to what magnitude serum lipase can increase in cats with subclinical histologically confirmed chronic pancreatitis. Our results suggest that the magnitude of lipase increase may be of little help, as cat 3 had the highest lipase values but remained clinically unaffected. Data on monitoring lipase values in cats over time are limited to one report measuring PLI in healthy kittens during their first year of life.^21^ The magnitude of lipase activities in the present study is comparable with reported lipase activities in hospitalised cats without a clinical diagnosis of pancreatitis.^10^ Our results provide an initial reference for clinicians regarding the possible magnitude of lipase activity and PLI increases, as well as their progression over time, in cats with a high likelihood of subclinical chronic pancreatitis.

The course of disease in cats 1 and 4 suggests that diarrhoea was caused by an enteropathy rather than pancreatitis. First, diarrhoea is a rare clinical sign in cats with pancreatitis.^3,10,22–24^ Second, both cats had an ultrasonographically normal pancreas; however, ultrasonographic (both cats) and endoscopic (cat 1) findings were consistent with intestinal disease. Third, we demonstrated that both diarrhoeic cats were diet-responsive and, most importantly, never exhibited common signs of feline pancreatitis, such as lethargy, inappetence or vomiting. Cat 1 underwent a full diagnostic workup, which revealed mild chronic inflammatory small bowel disease along with more severe chronic large bowel disease. Our results suggest that the fluctuating pattern of increased lipase activities and PLI concentrations is most likely due to subclinical chronic pancreatitis. Concurrent acute pancreatitis seems less likely, as cat 1 remained clinically stable for months while her intestinal disease was in remission, yet lipase values remained clearly increased.

All six cats had ultrasonographic evidence of enteropathy. A large-scale study reported a strong association between small bowel thickening >2.8 mm and intestinal disease in cats.^17^ Almost all cats (99/100) with small bowel segment thickening >2.8 mm had histological evidence of disease, both inflammatory and neoplastic.^17^ Cats often have concurrent chronic intestinal and pancreatic inflammatory disease,^9,10,25,26^ and it is also known that inflammatory lesions in both organs can be present without clinical signs.^25^ Although inflammation was documented in the upper and lower small bowel in cat 1, lesions were most severe in the large bowel, which matched the clinical picture. Large bowel diarrhoea has been reported to be more common in cats with diet-responsive enteropathy.^27^ The endoscopic and histopathological findings of diet-responsive enteropathy have not yet been reported. This cat finally responded to a hydrolysed diet (dry food 3) (Table 2). Cat 4 ultimately responded to a fibre-enriched diet (dry food 2) (Table 2). Dietary fat content appeared to have no impact on lipase and PLI levels or clinical status. The trial diet (dry food 2) contained higher total fat, primarily from animal sources, whereas the initial diet (dry food 1) had a lower total fat content (Table 2), most of it derived from plant sources. This supports previous findings that fat content alone does not significantly influence pancreatic enzyme activity in cats.^28–30^

At the time of writing (14 months since response to diets) both diet-responsive cats still have a stable body weight, good appetite and no other problems, except for occasionally slightly softer faeces. It is unknown whether treatments for intestinal disease also have a positive effect on the pancreas in cats. In cat 1, lipase values remained clearly increased, while in cat 4, lipase values increased again towards the end despite overall clinical improvement. Given the complete absence of clinical signs in four cats and improvement of the only clinical sign, diarrhoea, in two cats, it seems plausible that the cause of lipase increases in these cats was subclinical chronic pancreatitis.

Lipase values and pancreatic ultrasound findings did not align well in this small study, which reflects what has been reported previously.^4^ Ultrasonographic features of chronic pancreatitis are not well established in cats.^31,32^ It is generally assumed that cats with chronic pancreatitis have a hyper- or mixed-echoic or even normal pancreas, while a hypoechoic and enlarged pancreas is usually attributed to acute pancreatitis. Considerable overlap between these features has been reported,^3^ and it is possible that ultrasonography is not a suitable diagnostic tool for assessing chronicity. In our study, both cats with an ultrasonographically abnormal pancreas had a hypoechoic and enlarged pancreas in the absence of a hyperechoic mesentery. Both cats had clearly increased lipase levels within a range that is marketed as ‘consistent with pancreatitis’ for the PLI test,^33,34^ yet they exhibited no clinical signs at any time point. Without pancreatic histopathology, we cannot comment on acute vs chronic pancreatitis. However, the time course alone suggests a chronic process, and a hypoechoic, enlarged pancreas without hyperechoic mesentery may be seen in an acute flare-up with a subclinical course or chronic cases, further complicating the diagnostic interpretation.

It could be argued that colony cats from a research unit are not as closely monitored as privately owned cats and that more subtle signs of illness may have been missed. We argue that the situation of a private multi-cat household is not much different. We would like to point out that all cats are cared for daily by dedicated technicians in addition to being examined weekly by veterinarians. The clearest evidence of the cats’ clinical wellbeing, in addition to the absence of vomiting, inappetence, lethargy (all) or diarrhoea (n = 4/6), was their stable body weights. Subtle weight loss in otherwise normal-appearing pet cats often goes unnoticed by owners. We demonstrated stable body weight over the entire study period despite increased lipase levels.

Our study also has limitations. It would have been helpful to measure lipase levels in all cats at the same time points over a longer period. Simultaneous measurement of SAA concentrations at all time points could have helped to find out how acute and severe the inflammatory processes were. Histological examinations would have been ideal to characterise acute inflammation in the pancreas, but this examination is unfortunately far too invasive and was therefore never considered by the authors.

## Conclusions

This study shows the progression of lipase activity and PLI concentrations in clinically healthy cats and cats with diet-responsive enteropathy over time. The course and magnitude of lipase levels appeared erratic as it did not correlate with clinical or ultrasonographic findings. Cats can live normally with substantially increased lipases, making it challenging for clinicians to understand the significance of a lipase increase. Screening for concurrent extra-pancreatic disease that could also explain clinical signs is therefore important. Larger longitudinal studies are required to better understand the clinical relevance of lipase increases and potential dietary factors.
